# Genotype-phenotype correlation in a cohort of pediatric patients with autoinflammatory diseases carrying *NOD2* variants

**DOI:** 10.3389/fimmu.2025.1439333

**Published:** 2025-03-24

**Authors:** Marco Francesco Natale, Camilla Celani, Silvia Federici, Chiara Passarelli, Chiara Perrone, Emiliano Marasco, Fabrizio De Benedetti, Antonella Insalaco

**Affiliations:** ^1^ Division of Rheumatology, Bambino Gesù Children’s Hospital, IRCCS, Rome, Italy; ^2^ Laboratory of Medical Genetics, Translational Cytogenomics Research Unit, Bambino Gesù Children Hospital, IRCCS, Rome, Italy

**Keywords:** autoinflammatory diseases, *NOD2* gene, monogenic diseases, polygenic or complex inheritance, children

## Abstract

**Background:**

Autoinflammatory diseases (AIDs) are a group of disease characterized by excessive activation of the innate immune system with episodes of spontaneous inflammation that can affect different organs. Many monogenic or acquired autoinflammatory diseases are described in literature. More recently the concept of disease with polygenic or complex inheritance has been introduced. Nucleotide binding oligomerization domain containing 2 (NOD2) gene variants are associated with Crohn’s disease (CD), Blau syndrome and most recently with a polygenic autoinflammatory disease with onset in adult called NOD2-associated autoinflammatory disease (NAID).

**Objective:**

The aim of our study is to describe a pediatric cohort of patients with autoinflammatory disease carrying *NOD2* variants and to evaluate genotype-phenotype correlation.

**Methods:**

Twenty-five children with autoinflammatory disease and *NOD2* variants were enrolled in the study. Patients were divided into 3 groups based on the protein domain involved. Demographic and clinical features, imaging, laboratory exams and treatment were analyzed. The characteristics of our patients were compared with those of the adult cohort described by Yao in 2016-2018.

**Results:**

Fever was the main clinical characteristic of our children (68%) with long episodes and irregular pattern of recurrence. The disease typically affected skin (40%), joints (72%), bowel (60%) and lymphatic system (52%). Serositis and sensorineural deafness were less frequent. Excluding non-steroidal anti-inflammatory drugs (NSAIDs), glucocorticoids were frequently used with satisfactory clinical response in the majority of patients. In patients with poor disease control or new flares after glucocorticoid tapering, non-biologic and biologic drugs were used with variable response. The comparison between the two most represented groups showed that patients with variants located on the NOD domain presented more homogeneous clinical characteristics with involvement of some target organs. Our patients were compared with the adult cohort described in literature with few differences.

**Conclusion:**

This is the first study to evaluate genotypic/phenotypic characteristics of children with systemic autoinflammatory disease and *NOD2* variants. The results, albeit preliminary and affected by the sample size, do not allow a definitive conclusion on a monogenic disease caused by mutation in *NOD2*, with the obvious exception of Blau syndrome. Variants in the NOD domain seem to be associated with a more homogenous clinical phenotype.

## 1 Introduction

Autoinflammatory syndromes are a group of diseases characterized by excessive activation of the innate immune system with episodes of spontaneous inflammation that can affect several organs (1). Since 1997, when *MEFV* gene mutations were identified as causing Familial Mediterranean fever, dozens of new genes of autoinflammatory diseases (AIDs) have been identified. However, AIDs may also have a polygenic or multifactorial origin, with environmental factors modulating the phenotype (2). Nucleotide-binding oligomerization domain containing 2 (NOD2), also known as caspase recruitment domain-containing protein 15 (CARD15), is a protein encoded by the *NOD2* gene and belongs to the Nod-Like-Receptors’ (NLRs) group. In humans it plays a fundamental role in the immune system recognizing bacterial molecules (peptidoglycans) and stimulating the immune response (3). NOD2 interacts with several molecules and pathways, including receptor interacting protein kinase 2 (RIP2) and the interferon regulatory factor 4 (IRF4) pathway through cross-talk with Toll-like receptors, leading to NF-kB activation (4, 5). Given the complexity of these interactions, multiple functions of the protein and clinical correlates of the variants may be expected. This is consistent with the observations implicating multiple *NOD2* variants in diseases that are apparently very different: autoinflammatory diseases (monogenic and polygenic), inflammatory bowel diseases, cardiovascular diseases and cancer (6). NOD2 protein is composed by two tandem CARD domains that function as an effector and are activated by muramyl dipeptide, a bacterial cell wall fragment. On activation, NOD2 is believed to undergo self-oligomerization via the central NOD domain, providing a scaffold for recruiting RIPK2 (receptor-interacting serine–threonine kinase 2) through CARD–CARD interactions. Consequently, NOD2 stimulation results in activation of proinflammatory transcription factors including nuclear factor-κB (NF-κB), resulting in increased cytokine expression (7). *NOD2* sequence variants have been associated with Crohn’s disease (CD) (8), Blau syndrome (9–11), and more recently with an autoinflammatory disease with adult onset referred to as *NOD2* associated inflammatory diseases (NAID) or Yao’s syndrome (12, 13).

NAID is characterized by periodic fever, dermatitis, arthritis, gastrointestinal (GI) involvement and sicca-like symptoms (14). In 2016, a large cohort of adult patients with undefined AID showed a frequency of *NOD2* variants significantly higher than historical healthy controls, leading to the hypothesis of a role of *NOD2* variants in NAID (13). The largest cohort of patients with adult-onset autoinflammatory disease carrying *NOD2* variant has recently been described. Most of the patients, both with monogenic or digenic variants, were compound heterozygous for IVS8 + 158 and another one or more *NOD2* variants, such as R702W/SNP8, 1007fs, V955I or rare *NOD2* variants. A single heterozygous *NOD2* variant, such as IVS8 + 158, V955I or rare variants are most rarely described. A higher frequency of *NOD2* variants compared to healthy controls was confirmed. Patients carrying only *NOD2* variants were compared with patients with both *NOD2* and variants in other genes causing AID, (e.g. *MEFV*, *NLRP3*, *NLRP12* and *TNFRSF1A*), no significant differences in the clinical phenotypes were found, leading to the hypothesis that NOD2 variants were causative for the clinical phenotype (15).

We describe demographic, clinical, imaging and laboratory features and treatments of children with AID associated with *NOD2* variants.

## 2 Material and methods

### 2.1 Patients

**Figure 1:**
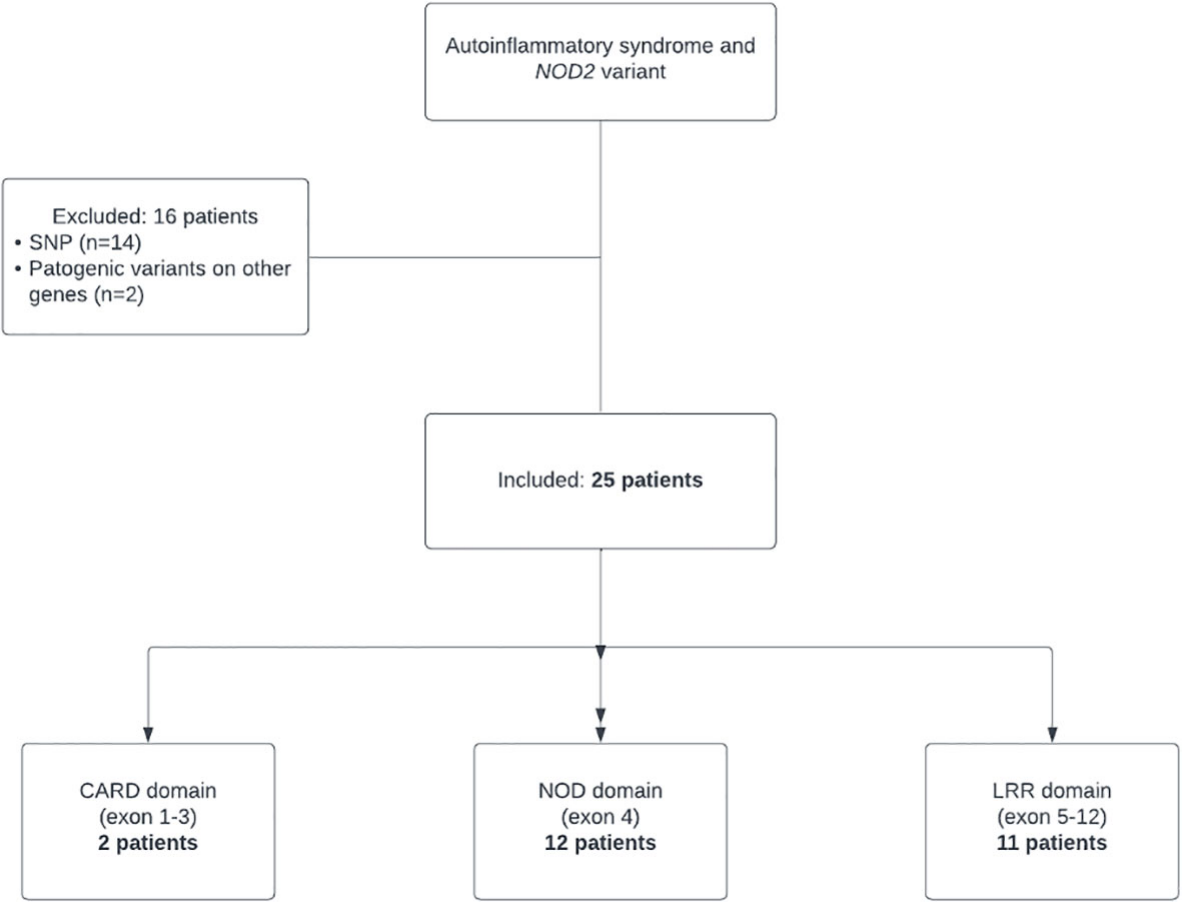
Flow chart for the study cohort.

A total of 41 children were recruited from our tertiary medical center. We selected patients with disease onset from 0 to 18 years of age with clinical, laboratory and features suggestive of autoinflammatory disease according to the following criteria: *NOD2* variants and recurrent course of the disease for at least one year, defined as self-limiting “inflammatory” flares lasting from few days to several weeks and with at least 2 of the following characteristics: fever, increase of inflammatory markers (CRP > 2ULN/ESR >2 ULN), skin rash, gastrointestinal involvement (diarrhea and/or vomit and/or abdominal pain and/or stool blood), arthralgia/arthritis and inflammatory bone lesions radiologically confirmed. Patients were excluded if they had variants on *NOD2* classified as pathogenic for Blau syndrome, variants of other genes classified as pathogenic for a monogenic AID or variants on *NOD2* classified as single nucleotide polymorphism (SNP) very common in healthy population (**Figure 1**).

For each patient the following data were collected from the electronic databases of our Hospital: gender, age at onset, age at diagnosis, comorbidities, symptoms and clinical features, laboratory and imaging and treatments. For the genetics analysis, probands and, when available, parents’ genomic DNA was extracted from peripheral blood and analyzed through Next Generation Sequencing. Therapeutic choices and their efficacy were evaluated for each patient. Complete response was defined as >80% reduction of the number of inflammatory flare/year or complete resolution of the organ lesions. Partial response was defined as >50% reduction of the number of inflammatory flare/year or partial resolution of the organ lesions. No response was defined as absence of partial response.

### 2.2 Genetic testing

Genetic tests using an NGS (Next Generation Sequencing) targeted-resequencing panel including genes related with autoinflammatory diseases or a “clinical exome” panel (T6920 genes known as associated to genetic disorders) were performed on all patients on the Illumina NovaSeq6000 platform. The reads were aligned to human genome build GRCh37/UCSC hg19. The BaseSpace pipeline and the Geneyx software LifeMap Sciences were respectively used for the variant calling and annotating variants. For the “clinical exome” analysis, the variants were filtered by *in silico* analysis on genes associated to autoinflammatory diseases. Global minor allele frequency (MAF) for analyzed variants was calculated according to Genome Aggregation Database (gnomAD). The variants were evaluated by VarSome and in accordance with the American College of Medical Genetics and Genomics (ACMG) recommendations (16, 17). Variants were also examined for coverage and Qscore (minimum threshold of 30) and visualized by the Integrative Genome Viewer (IGV).

### 2.3 Statistical analysis

For descriptive statistics, continuous variables were expressed as median and range and categorical variables were presented as frequency or percentage. Chi-square test or Fisher test, as appropriate, were used to compute the differences in frequency between subgroups and between our pediatric cohort and the adult cohort reported in literature. Multiple Correspondence Analysis (MCA) was used to evaluate the homogeneity/heterogeneity of phenotypic expression within the subgroups (i.e. protein domain). All tests were two-sided and a p-value <0.05 was considered statistically significant.

## 3 Results

### 3.1 Patients

**Figure 2:**
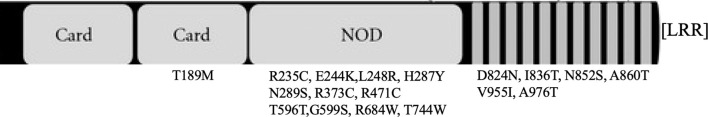
Localization of the variants in the domain of the NOD2 protein (CARD/NOD/LRR).

We analyzed 41 patients with AID according to the inclusion criteria. Fourteen patients were excluded because of the presence of single nucleotide polymorphisms reported with high frequency in the general population (P268S/SNP5, R702W/SNP8, G908R/SNP12 and 1007FS/SNP13). One patient carrying the S178S NOD2 variant was excluded because of the presence of M694V homozygosity in the MEFV gene, therefore diagnosed with FMF, and one patient carrying the L248R NOD2 variant was excluded because of the presence of F508S hemizygosity in the TLR7 gene therefore diagnosed with monogenic lupus. Finally, 25 patients were analyzed. They were subsequently divided into 3 subgroups based on the localization of the NOD2 variants. This subgroup division is based on the different roles played by NOD2 domains in inflammatory pathways. The leucine-rich repeat (LRR) domain plays a role in the recognizing of microbial components and regulates NOD2 dimerization. The caspase recruitment domain (CARD) binds and activate caspase-1 and has a role in IL-1β processing. The nucleotide-binding and oligomerization domain (NOD) regulates the oligomerization of NOD2 induced by ATP and promotes the activation of the NF-kB pathway. LRR variants are implicated in the susceptibility to Crohn’s disease with a loss of function mechanism; NOD variants cause Blau syndrome with a gain of function mechanism (4, 5). This is related with *NOD2* role as activator of the NF-kB inflammatory pathway. In Blau patients (gain of function) it is hyperactive, while in Crohn’s disease being defective (loss of function). This results in a defect in the normal inflammatory response to intestinal bacteria, thereby leading to the hypothesis of a greater susceptibility to the development of IBD. **Figure 2** shows all variants detected in the analyzed patients and their localization on the NOD2 protein. We identified four novel variants. Two in the NOD group (E244K and G599S) and two in the LRR group (A860T and A976T). All variants were classified as variants of uncertain significance.

Of the 25 patients included, all Caucasians, 14 were males (56%). The median age at presentation was 2.6 years (range 0.3 – 17 years). A complete family history was available in 26 patients. In one male patient (4%) a family history (father, one brother and one sister) of a disease with an autoinflammatory phenotype was referred: the father had recurrent fever and skin rash, the patient’s sister recurrent fever, skin rash, oral and genital aphtosis and bowel inflammation, and the patient’s brother had bone lesions and skin involvement consistent with synovitis-acne-pustulosis-hyperostosis-osteitis syndrome (SAPHO).

### 3.2 Clinical phenotype

Clinical features are described in **Table 1**. Fever was the main clinical characteristic of our cohort with 17 patients (68%) presenting recurrent febrile episodes with irregular pattern of recurrence. Patients presented a median of 12 febrile episodes/year (range 3-24). Episodes lasted 3-7 days in the majority and more than 7 days in 27.7%.

**Table 1:** Features of patients with autoinflammatory syndrome carrying NOD2 variants.

	Total (n=25*)	NOD (n=12)	LRR (n=11)	P-Value
Demographic Characteristics				
Gender (male)	14 (56%)	6 (60%)	7 (63.6%)	>0.2
Family history of autoinflammatory disease (yes)	1 (4%)	1 (8%)	0	>0.2
Consanguinity	0	0	0	
Ethnicity (Caucasian)	25 (100%)	12 (100%)	11 (100%)	>0.2
Clinical Characteristics				
Skin and mucosal involvement	21 (84%)	11 (91%)	8 (72%)	>0.2
- Oral Aphthosis	14 (56%)	7 (58%)	6 (54%)	>0.2
- Urticaria	6 (24%)	4 (33%)	2 (18%)	>0.2
- Maculo-papular rash	4 (16%)	2 (16%)	1 (9%)	>0.2
- Pharyngitis	8 (32%)	6 (50%)	2 (18%)	0.19
- Psoriasis	1 (4%)	1 (8%)	0	>0.2
- Other **	3 (12%)	0	2 (18%)	>0.2
Osteoarticular involvement	18 (72%)	7 (58%)	9 (81%)	>0.2
- Arthralgia	18 (72%)	7 (58%)	9 (81%)	>0.2
- Arthritis	7 (28%)	1 (8%)	5 (45%)	0.06
- Bone inflammation	4 (16%)	0	4 (36%)	0.03
Gastrointestinal involvement	15 (60%)	7 (58%)	8 (72%)	>0.2
- Abdominal Pain	14 (56%)	7 (58%)	7 (63%)	>0.2
- Vomit	4 (16%)	2 (16%)	2 (18%)	>0.2
- Diarrhea	7 (28%)	5 (41%)	2 (18%)	>0.2
- Gastrointestinal bleeding	1 (4%)	0	1 (9%)	>0.2
- Other***	2 (8%)	0	2 (18%)	>0.2
Lymphatic system involvement	13 (52%)	6 (50%)	5 (45%)	>0.2
- Diffuse Lymphadenopathies	4 (16%)	1 (8%)	2 (18%)	>0.2
- Cervical Lymphadenopathies	9 (36%)	5 (41%)	3 (27%)	>0.2
- Liver enlargement	4 (16%)	1 (8%)	2 (18%)	>0.2
- Spleen enlargement	3 (12%)	0	2 (18%)	>0.2
- Spleen focal lesions	1 (4%)	0	1 (9%)	>0.2
Other				
- Serositis	3 (12%)	0	2 (18%)	>0.2
- Recurrent epididymitis	1 (4%)	0	1 (9%)	>0.2
- Amyloidosis	0	0	0	>0.2
- HLH/MAS	2 (8%)	2 (16%)	0	>0.2
- CNS involvement	1 (4%)	0	1 (9%)	>0.2
Laboratory and imaging				
- CRP increase	14 (56%)	5 (41%)	8 (72%)	>0.2
- ANA	2 (8%)	0	1 (9%)	>0.2

*Two patients with a variant in N-term/CARD domain are excluded from this analysis.

** Acne and acrocyanosis.

***Acute pancreatitis or constipation.

The scale of greens identifies the percentages <25%, 25-75% and >75%. In “p-value” section, red indicates statistically significant values (p<0.05) while yellow indicates non-significant values.

Skin and mucosal involvement was present in 21 patients (84%) with recurrent episodes of maculopapular skin rash, urticaria and minor oral aphthosis. Giant oral ulcers were reported rarely (2 patients). Eighteen patients (72%) presented with joint pain and 7 with overt transient arthritis (non-erosive). Gastrointestinal involvement with diarrhea/vomit or abdominal pain was observed in 60% of the patients. Lymphadenopathies and hepatomegaly or splenomegaly in 52%.

Serositis was reported in 3 patients (1 pericarditis, 1 pleuritis and 1 pleuropericarditis).

Severe sensorineural deafness was observed in only one patient with a unique phenotype characterized by serositis, bone osteolytic lesions, acne, bowel inflammation and interstitial lung disease. Increased inflammatory markers during flares were detected in the majority (56%). Only two patients had antinuclear antibodies (ANA) at low titer (1:160, speckled pattern). Lymphocyte subsets, including natural killer, T cells and B cells absolute numbers and serum immunoglobulin levels were normal in all patients. Whole-body Magnetic Resonance Imaging (MRI) and/or bone scintigraphy were performed in 10 patients, (because of joint/bone pain) and showed bone inflammatory involvement in 4 patients.

In 6 of the 15 children with gastrointestinal symptoms endoscopy, consisting of colonoscopy and esophagogastroduodenoscopy, was performed. In the patient with sensorineural deafness it revealed diffuse aphthosis of the duodenum with colic infiltrate composed by lymphocytes, plasmacells and eosinophils. In the other 5 patients no macroscopic signs of mucosal inflammation were observed. Histology showed in 3 of these 5 cases mild inflammatory infiltrate (nodular lymphoid hyperplasia and slight increase of the eosinophilic and granulocyte count) which was deemed not suggestive of a classical inflammatory bowel disease (IBD).

### 3.3 Treatments

Treatment of our patients was empiric and based on clinical features (**Table 2**). Glucocorticoids were frequently used (methylprednisolone or betamethasone) with satisfactory clinical response in the majority of the patients. Short courses were used, with none of the patients requiring chronic glucocorticoid treatment.

**Table 2:** Treatments used in NOD2 carrier patients and their efficacy.

Drugs	No response	Partial Response	Complete Response
Colchicine (n= 9)	2/9 (22%)	5/9 (55%)	2/9 (22%)
Other* (n = 3)	1/3 (33%)	1/3 (33%)	1/3 (33%)
IL-1 inhibitors (n = 7)	1/7 (14%)	1/7 (14%)	5 (71%)
IL-6 inhibitor (n = 2)	0/2 (0%)	0/2 (0%)	2/2 (100%)
TNF inhibitors (n = 2)	0/2 (0%)	1/2 (50%)	½ (50%)

Complete response was defined as >80% reduction of the number of inflammatory flare/year or complete resolution of the organ lesions.

Partial response was defined as >50% reduction of the number of inflammatory flare/year or partial resolution of the organ lesions.

No response was defined as absence of partial response.

Refer to **Supplementary Table 1** for analysis of therapeutic response in subgroups.

*Cyclosporine A (n = 1) or Methotrexate (n= 2).

In patients with poor disease control or new flares after glucocorticoid tapering, non-biologic and biologic drugs were used. Colchicine was used in 9 patients of whom 2 had complete response and 5 partial response. Seven patients received IL-1 inhibitors (anakinra and canakinumab). Most had a complete or partial response (85%) with one therapeutic failure. IL-6 inhibitor (tocilizumab) was used in two patients with good response. TNF alfa inhibitor (etanercept) was used in 2 patients with 1 partial and 1 complete response. DMARDs (Cyclosporine A (n = 1) or Methotrexate (n = 2)) were rarely used with inconsistent efficacy.

### 3.4 Clinical phenotypes of the NOD group and the LRR group

We compared the clinical features of patients carrying a variant in the NOD domain (NOD group) with those of patients carrying a variant in the LRR domain (LRR group). The two patients with a variant in N-term/CARD domain were excluded from this analysis: these patients presented a phenotype characterized by recurrent fever, oral aphthous, skin rash, arthritis and abdominal pain, with no distinctive features. Patients in the NOD group showed a homogeneous clinical phenotype with prevalent involvement of skin (urticaria and macular rash), oral mucosa (aphthosis and pharyngitis), osteoarticular system, gastrointestinal tract and lymphatic organs. Patients in the LRR group presented with more heterogeneous manifestations with scattered heterogenous features such as variable skin involvement (acne and acrocyanosis) sacroiliitis, pancreatitis, focal lesions of the spleen and sensorineural deafness. Bone inflammatory lesions suggestive of chronic nonbacterial osteomyelitis (CNO) were detected in 4 patients in the LRR group and in none of the NOD group. Overt transient arthritis was observed in 5 patients of the LRR group and in 1 of the NOD group. We analyzed the clinical differences between the two groups through a Multiple Correspondence Analysis (MCA). The first four dimensions captured jointly around 58% of variance. Dimension 1 (21% of variance) was driven by serositis and gastrointestinal bleeding, dimension 2 (15.3% of variance) was mainly driven by mucocutaneus items.

**Figure 3:**
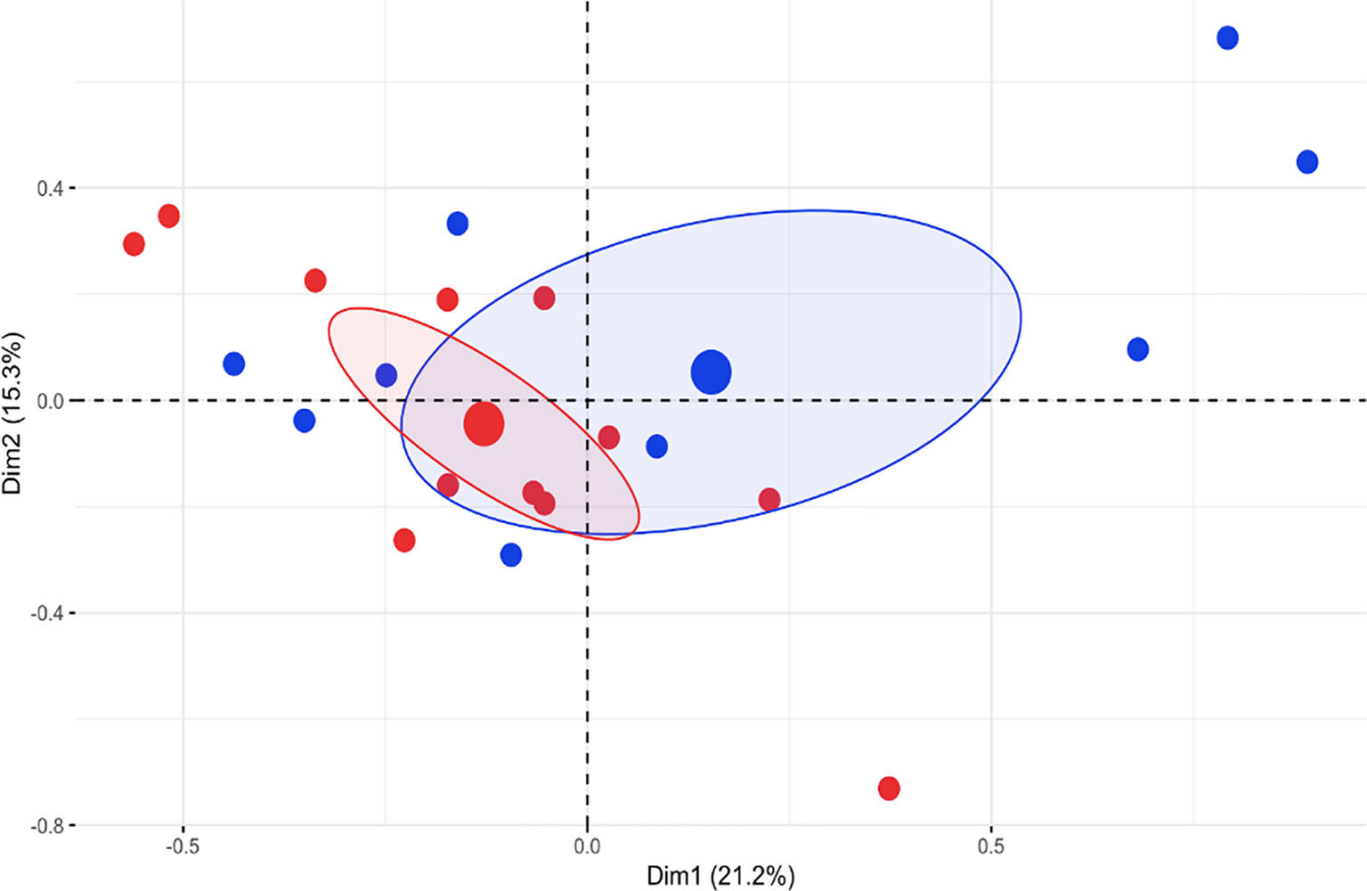
Multiple Correspondence Analysis (MCA) of the first 2 dimensions (Dim) of clinical data (% variance explained is indicated). Blue dots represent patients of the LLR group, red dots represent patients of the NOD group. Big dots represent epicenters of distributions and ellipses indicate 95% confidence intervals.

Except for a slight difference along the first dimension, a clear separation between the two groups was not detected (**Figure 3**). No significant difference in treatment response was found between two groups (**Supplementary Table 1**).

### 3.5 Differences between pediatric and adult patients carrying a *NOD2* variant

We compared the features of the children reported here with the adult cohort previously described (8). A difference in the gender prevalence was detected with more female patients in the adult cohort (M/F ratio 1:2) compared to our pediatric cohort (M/F Ratio 1.3:1). In both groups, family history consistent with autoinflammatory diseases was rare (4% vs 7%).

Recurrent fever was the main clinical feature for both (68% vs 61%). The most frequently involved organs were skin (40% vs 91%) joints (72% vs 87%) and bowel (60% vs 72%). Autoantibodies were rare in both groups.

Significant differences were found in skin rash (40% vs 91%) and arthritis (28% vs 85%), which were more common in the adult cohort. Lymphatic system involvement (52% vs 9%) and oral aphthosis (56% vs 26%) were more frequent in children. (**Table 3**) In both cohorts, gastrointestinal endoscopy, when performed, showed normal mucosal appearance with mild and non-specific inflammation at histology that was not classified as IBD.

**Table 3:** Features of our pediatric cohort were compared with the adult cohort reported in the literature (8).

	Children (n=25)	Adults (n=54)	P-value
Female	11 (44%)	37 (69%)	0.03
Family History	1 (4%)	4 (7%)	>0.2
Fever	17 (68%)	33 (61%)	>0.2
Skin rash	10 (40%)	49 (91%)	< 0.01
Oral aphthosis	14 (56%)	14 (26%)	< 0.01
Myalgia	8 (32%)	19 (35%)	>0.2
Arthralgia/Arthritis	18 (72%)	47 (87%)	0.1
Arthritis	7 (28%)	46 (85%)	< 0.01
Lymphadenopathies	13 (52%)	5 (9%)	< 0.01
Sicca Syndrome	0 (0%)	30 (56%)	n.a.
Bowel symptoms	15 (60%)	39 (72%)	>0.2
Endoscopy *	6/15 with bowel symptoms	n.a.	
Inflammatory infiltrate at histology	4 (16%)	4 (7%)	>0.2
Serositis	3 (12%)	5 (9%)	>0.2
Sensorineural deafness	1 (4%)	0 (0%)	n.a.
CRP increase	14 (56%)	21 (39%)	0.15
ANA +	2 (8%)	4 (7%)	>0.2

* Endoscopy was performed in 6 children with gastrointestinal symptoms.

NA, not applicable.

Similar data were obtained comparing treatments. In adult patients with persistent inflammation and/or incomplete response to glucocorticoids, biologics were used with the best efficacy reported for IL-1 inhibitors (18).

## 4 Discussion

The aim of our work was to evaluate the clinical phenotype and the genotype-phenotype correlation in a pediatric cohort of patients with AID carrying a *NOD2* variant.

In an attempt to analyze the potential clinical relevance of *NOD2* variants, we have chosen to exclude patients carrying variants known to be pathogenic for Blau syndrome or carrying SNP reported as very frequent in healthy population, including those associated with Crohn’s disease. We analyzed 25 patients with an AID phenotype. Their clinical phenotype was markedly heterogenous making it difficult to identify a unique syndrome associated with these variants. In order to better understand the potential role of different genetic variants in *NOD2*, patients were divided into three groups based on the protein domain involved. Only two patients carried a variant in the CARD domain and these were excluded from this comparative analysis. Eventually, 12 patients carrying a variant in the NOD domain and 11 carrying a variant in LRR domain were compared. Our analysis showed homogeneity within the NOD group suggesting a possible greater “weight” of these variants as risk factors for disease development. Indeed, there were some remarkable differences in the frequency of manifestations among the two groups, with arthritis being more frequent in the LRR group and inflammatory bone lesion and serositis being observed only in the same group. When MCA was used a clear distinction between the two groups was not defined but patients of the NOD group showed more homogeneous phenotypes. This could suggest that variants in the NOD domain may underlie a definable clinical syndrome. However, a much larger sample size is needed to support this conclusion.

In attempt to further analyze the issue we have tried to use the data of the largest series of adult patients available in the literature (13). It should be mentioned that, in the series of adult patients, Blau syndrome causing variants were excluded. However, three of the variants included in the adult study were excluded from our study because they are reported as single nucleotide polymorphisms (G908R, R702W and 1007fs). Despite this limitation, comparison of our pediatric series with their series showed some differences, with the main being a higher frequency of joint involvement in adults and a higher frequency of oral aphthosis in children. However, it should be noted that, in addition to the above-mentioned inclusion of polymorphisms, adult patients were not divided according to the domain involved.

At present, the available data do not allow a definitive conclusion on the presence of a monogenic disease caused by variants in *NOD2*, with the obvious exception of Blau syndrome. It is possible that variants in *NOD2* could play a role as susceptibility factors for an AID with polygenic or complex genetic inheritance. Indeed, as it is often the case for diseases with complex genetic inheritance, we did not identify a clear unique genotype/phenotype correlation. Moreover, family history was present only in 1 of the pediatric and 4 of the adult patients, in contrast with a classical dominant model. Nevertheless, the phenotypic homogeneity identified by MCA suggests that variants in the NOD domain may have an impact on clinical expression. Recently, another study analyzed the potential effect of *NOD2* variants on the clinical phenotypes in patients with NAID or other monogenic (FMF) or polygenic autoinflammatory disease. In their cohort patients with NAID and those with other diagnoses shared common variants. This observation points towards the hypothesis that carriage of specific *NOD2* variants may confer susceptibility to disease by modifying or amplifying inflammation rather than be the direct cause of a specific disease (19).

In conclusion, this is the first study that evaluated the genotypic/phenotypic correlation in children with AID carrying NOD2 variants. Our results, albeit limited by the sample size, together with those obtained in a large adult cohort, do not allow to identify a unique clinical syndrome caused by NOD2 variants. Therefore, presence of one of these variants in patients with an undefined autoinflammatory disease should not distract from the search for other underlying causes of the disease.

The observation that variants in the NOD domain are associated with at least some phenotypic homogeneity requires further studies on much larger series of patients, ideally multicenter and in children. These studies should rigorously exclude polymorphisms. The differences between pediatric and adult patients, in gender prevalence as well as clinical phenotype, may also suggest a potential hormonal role in the multifactorial pathogenesis of this condition. Further studies may clarify this aspect as well.

## Data Availability

The raw data supporting the conclusions of this article will be made available by the authors, without undue reservation.

## References

[1] Zen M Gatto M Domeneghetti M Palma L Borella E Iaccarino L. Clinical guidelines and definitions of autoinflammatory diseases: contrasts and comparisons with autoimmunity-a comprehensive review. Clin Rev Allergy Immunol. (2013) 45:227–35. doi: 10.1007/s12016-013-8355-1 23322404

[2] Krainer J Siebenhandl S Weinhäusel A. Systemic autoinflammatory diseases. J Autoimmun. (2020) 109:102421. doi: 10.1016/j.jaut.2020.102421 32019685 PMC7610735

[3] Mahla RS Reddy MC Prasad DV Kumar H. Sweeten PAMPs: role of sugar complexed PAMPs in innate immunity and vaccine biology. Front Immunol. (2013) 4:248. doi: 10.3389/fimmu.2013.00248 24032031 PMC3759294

[4] Yao Q. Nucleotide-binding oligomerization domain containing 2: structure, function, and diseases. Semin Arthritis Rheum. (2013) 43:125–30. doi: 10.1016/j.semarthrit.2012.12.005 23352252

[5] Watanabe T Minaga K Kamata K Sakurai T Komeda Y Nagai T. RICK/RIP2 is a NOD2-independent nodal point of gut inflammation. Int Immunol. (2019) 31:669–83. doi: 10.1093/intimm/dxz045 PMC6939834 31132297

[6] Negroni A Pierdomenico M Cucchiara S Stronati L. NOD2 and inflammation: current insights. J Inflammation Res. (2018) 11:49–60. doi: 10.2147/JIR.S137606 PMC5813767 29483781

[7] Maekawa S Ohto U Shibata T Miyake K Shimizu T. Crystal structure of NOD2 and its implications in human disease. Nat Commun. (2016) 7:11813. doi: 10.1038/ncomms11813 27283905 PMC4906405

[8] Hugot JP Chamaillard M Zouali H Lesage S Cézard JP Belaiche J. Association of NOD2 leucine-rich repeat variants with susceptibility to Crohn’s disease. Nature. (2001) 411(6837):599–603. doi: 10.1038/35079107 11385576

[9] Miceli-Richard C Lesage S Rybojad M Prieur AM Manouvrier-Hanu S Häfner R. CARD15 mutations in Blau syndrome. Nat Genet. (2001) 29(1):19–20. doi: 10.1038/ng720 11528384

[10] Wouters CH Maes A Foley KP Bertin J Rose CD. Blau syndrome, the prototypic auto-inflammatory granulomatous disease. Pediatr Rheumatol Online J. (2014) 12:33. doi: 10.1186/1546-0096-12-33 25136265 PMC4136643

[11] Kanazawa N Okafuji I Kambe N Nishikomori R Nakata-Hizume M Nagai S. Early-onset sarcoidosis and CARD15 mutations with constitutive nuclear factor-kappaB activation: common genetic etiology with Blau syndrome. Blood. (2005) 105:1195–7. doi: 10.1182/blood-2004-07-2972 15459013

[12] Yao Q Zhou L Cusumano P Bose N Piliang M Jayakar B. A new category of autoinflammatory disease associated with NOD2 gene mutations. Arthritis Res Ther. (2011) 13:R148. doi: 10.1186/ar3462 21914217 PMC3308076

[13] Yao Q Shen M McDonald C Lacbawan F Moran R Shen B. NOD2-associated autoinflammatory disease: a large cohort study. Rheumatol (Oxford). (2015) 54:1904–12. doi: 10.1093/rheumatology/kev207 26070941

[14] Yao Q Su LC Tomecki KJ Zhou L Jayakar B Shen B. Dermatitis as a characteristic phenotype of a new autoinflammatory disease associated with NOD2 mutations. J Am Acad Dermatol. (2013) 68:624–31. doi: 10.1016/j.jaad.2012.09.025 23102769

[15] Nomani H Deng Z Navetta-Modro1 B Yang J Yun M Aroniadis O. Implication of combined NOD2 and other gene mutations in autoinflammatory diseases. Front Immunol. (2023) 14. doi: 10.3389/fimmu.2023.1265404 PMC10620916 37928541

[16] Kopanos C Tsiolkas V Kouris A Chapple CE Albarca Aguilera M Meyer R. VarSome: the human genomic variant search engine. Bioinformatics. (2019) 35:1978–80. doi: 10.1093/bioinformatics/bty897 PMC6546127 30376034

[17] Richards S Aziz N Bale S Bick D Das S Gastier-Foster J. Standards and guidelines for the interpretation of sequence variants: a joint consensus recommendation of the American College of Medical Genetics and Genomics and the Association for Molecular Pathology. Genet Med. (2015) 17:405–24. doi: 10.1038/gim.2015.30 PMC4544753 25741868

[18] Yao Q Shen B. A systematic analysis of treatment and out- comes of NOD2-associated autoinflammatory disease. Am J Med. (2017) 130:365.e13–e18. doi: 10.1016/j.amjmed.2016.09.028 27984003

[19] Karamanakos A Vougiouka O Sapountzi E Venetsanopoulou AI Tektonidou MG Germenis AE. The expanding clinical spectrum of autoinflammatory diseases with NOD2 variants: a case series and literature review. Front Immunol. (2024) 15:1342668. doi: 10.3389/fimmu.2024.1342668 38348033 PMC10859468

