# Supplementary material for: Genotype-phenotype correlation in a cohort of pediatric patients with autoinflammatory diseases carrying NOD2 variants
Source: Front Immunol. 2025 Mar 24;16:1439333. doi: 10.3389/fimmu.2025.1439333 (PMC11973280; doi:10.3389/fimmu.2025.1439333)
Supplement: Supplementary file 1 [file Table1.docx]

SUPPLEMENTARY TABLE 1

Comparison of the treatment between the two most relevant groups

| Drugs | NOD (n=12) | LRR (n=11) | P-Value |
| --- | --- | --- | --- |
| Colchicine (n= 9) | 4/12 (33%) | 4/11 (36%) | >0.2 |
| No response | 1/4 (25%) | 1/4 (25%) | >0.2 |
| Partial Response | 1/4 (25%) | 3/4 (75%) | >0.2 |
| Complete Response | 2/4 (50%) | 0/4 (0%) | >0.2 |
| IL-1 inhibitors (n = 7) | 3/12 (25%) | 4/11 (36%) | >0.2 |
| No response | 0/3 (0%) | 1/4 (25%) | >0.2 |
| Partial Response | 0/3 (0%) | 1/4 (25%) | >0.2 |
| Complete Response | 3/3 (100%) | 2/4 (50%) | >0.2 |
| IL-6 inhibitors (n = 2) | 0/12 (0%) | 1/11 (9%) | >0.2 |
| No response | 0/0 (0%) | 0/1 (0%) | >0.2 |
| Partial Response | 0/0 (0%) | 0/1 (0%) | >0.2 |
| Complete Response | 0/0 (0%) | 1/1 (100%) | >0.2 |
| TNF inhibitors (n = 2) | 0/2 (0%) | 2/11 (18%) | >0.2 |
| No response | 0/0 (0%) | 0/2 (0%) | >0.2 |
| Partial Response | 0/0 (0%) | 1/2 (50%) | >0.2 |
| Complete Response | 0/0 (0%) | 1/2 (50%) | >0.2 |

*Two patients with a variant in N-term/CARD domain are excluded from this analysis. One patient was treated with colchicine with partial response. The other was treated with tocilizumab with good response.
