# Supplementary material for: Genotype-phenotype correlation in a cohort of pediatric patients with autoinflammatory diseases carrying NOD2 variants
Source: Front Immunol. 2025 Mar 24;16:1439333. doi: 10.3389/fimmu.2025.1439333 (PMC11973280; doi:10.3389/fimmu.2025.1439333)
Supplement: Supplementary file 2 [file Table2.docx]

SUPPLEMENTARY TABLE 2

Genomic data of the patients

| Patient n° | Genetic variant | Group | rs | ClinVar ID |
| --- | --- | --- | --- | --- |
| 1 | T189M | CARD | rs61755182 | 319431 |
| 2 | T189M | CARD | rs61755182 | 319431 |
| 3 | R235C | NOD | rs104895422 | 97879 |
| 4 | R235C | NOD | rs104895422 | 97879 |
| 5 | E244L | NOD | rs1964420065 | 1694117 |
| 6 | L248R | NOD | rs104895423 | 97881 |
| 7 | H287Y | NOD | rs560242309 | 319439 |
| 8 | N289S | NOD | rs5743271 | 319440 |
| 9 | R373C | NOD | rs145293873 | 97902 |
| 10 | R471C | NOD | rs1078327 | 319446 |
| 11 | G559S | NOD | rs369310865 | SUB15171270 |
| 12 | T596T | NOD | rs104895437 | 97839 |
| 13 | R684W | NOD | rs5743276 | 319458 |
| 14 | R744W | NOD | rs140876663 | 653293 |
| 15 | D824N | LRR | rs61755272 | 734538 |
| 16 | D824N | LRR | rs61755272 | 734538 |
| 17 | I836T | LRR | rs763192145 | 1432593 |
| 18 | N852S | LRR | rs104895467 | 97856 |
| 19 | N852S | LRR | rs104895467 | 97856 |
| 20 | N852S | LRR | rs104895467 | 97856 |
| 21 | N852S | LRR | rs104895467 | 97856 |
| 22 | A860T | LRR | rs754270929 | 661846 |
| 23 | V955I | LRR | rs5743291 | 97869 |
| 24 | V955I | LRR | rs5743291 | 97869 |
| 25 | A976T | LRR | rs148561632 | 531609 |
